# Population Based Testing for Primary Prevention: A Systematic Review

**DOI:** 10.3390/cancers10110424

**Published:** 2018-11-05

**Authors:** Ranjit Manchanda, Faiza Gaba

**Affiliations:** 1 Barts Cancer Institute, Queen Mary University of London, Old Anatomy Building, Charterhouse Square, London EC1M 6BQ, UK; f.gaba@qmul.ac.uk; 2 Department of Gynaecological Oncology, St Bartholomew’s Hospital, London EC1A 7BE, UK; 3 Gynaecological Cancer Research Centre, Department of Women’s Cancer, Institute for Women’s Health, University College London, 149 Tottenham Court Road, London W1T 7DN, UK

**Keywords:** population testing, genetic testing, BRCA, Jewish, general population, cancer prevention, primary prevention

## Abstract

The current clinical model for genetic testing is based on clinical-criteria/family-history (FH) and a pre-defined mutation probability threshold. It requires people to develop cancer before identifying unaffected individuals in the family to target prevention. This process is inefficient, resource intensive and misses >50% of individuals or mutation carriers at risk. Population genetic-testing can overcome these limitations. It is technically feasible to test populations on a large scale; genetic-testing costs are falling and acceptability and awareness are rising. MEDLINE, EMBASE, Pubmed, CINAHL and PsychINFO databases were searched using free-text and MeSH terms; retrieved reference lists of publications were screened; additionally, web-based platforms, Google, and clinical-trial registries were searched. Quality of studies was evaluated using appropriate check-lists. A number of studies have evaluated population-based *BRCA*-testing in the Jewish population. This has been found to be acceptable, feasible, clinically-effective, safe, associated with high satisfaction rates and extremely cost-effective. Data support change in guidelines for population-based *BRCA*-testing in the Jewish population. Population panel testing for *BRCA1/BRCA2/RAD51C/RAD51D/BRIP1/PALB2* gene mutations is the most cost-effective genetic-testing strategy in general-population women and can prevent thousands more breast and ovarian cancers than current clinical-criteria based approaches. A few ongoing studies are evaluating population-based genetic-testing for multiple cancer susceptibility genes in the general population but more implementation studies are needed. A future population-testing programme could also target other chronic diseases.

## 1. Introduction

A number of moderate to high penetrance cancer-susceptibility genes (CSG) with well-established clinical utility have been identified over the last two decades, and testing for these is widely available in clinical practice. The prime, most well-known exemplars have been *BRCA1* and *BRCA2*. *BRCA1/BRCA2* carriers have a 17–44% risk of ovarian cancer (OC) and 69–72% risk of breast cancer (BC) until age 80 years [1]. The current model for genetic testing is still predominantly driven by family-history (FH) or clinical-criteria with testing undertaken in hospitals or specialist genetic clinics following informed pre-test counselling. These FH-based criteria have been used to calculate mutation probability with genetic testing offered over a pre-defined probability threshold. Clinical-criteria have been loosened and this threshold for offering testing has fallen over the years (from an earlier high of 20%), with most countries/health systems now offering *BRCA*-testing at about a 10% *BRCA*-mutation probability. A number of different models, ranging from standardized criteria to complex mathematical (Empirical/Mendelian) methodologies have been used to calculate mutation probability and are used in clinical practice. Carrier identification has numerous potential clinical benefits, which have been the main drivers for genetic testing. Effective options for prevention and/or screening are well-established for these mutation-carriers in clinical practice. Unaffected *BRCA*-mutation carriers can opt for risk-reducing salpingo-oophorectomy (RRSO) to reduce their OC-risk [2], as well as Magnetic Resonance Imaging (MRI)/mammography screening, and chemoprevention with selective estrogen-receptor-modulators (SERM) [3] or risk-reducing mastectomy (RRM) [4] to reduce their BC-risk. Additionally, mutation identification enables informed reproductive and contraceptive choices which can impact risk, including timing of pill use and planning of a family, as well as prenatal and pre-implantation genetic-diagnosis (PGD) [5]. Cancer-affected carriers may be eligible for treatment with novel drugs like Poly ADP ribose polymerase (PARP) inhibitors, which have been shown to improve survival in platinum sensitive recurrent disease (HR ranging from 0.18 to 0.35) [6], and may also be able to gain access to newer precision medicine based targeted therapeutics through clinical trials [7,8,9].

Pre-test genetic-counselling is a fundamental element of international guidelines [10] for informed decision-making before genetic-testing. The model for counselling has evolved over the years, with the original Huntingdon Model involving a minimum of two 60 min face-to-face pre-test counselling sessions [11] now archived as a fixture of the past. Telephone counselling, DVD-assisted and group-based approaches have been found to be non-inferior to traditional 1:1 face-to-face counselling [12,13,14,15,16,17]. Over the years, a wide variety of decision aids have been used as adjuncts to help informed decision making, such as booklets, pamphlets, audiotapes, computer-based programmes and web-based platforms. Another important recent development is the move away from traditional genetics clinics towards non-genetic clinicians undertaking routine pre-test counselling and testing at cancer diagnosis [18]. Nevertheless, the process still involves post-test counseling by genetic counselors/clinicians, and is also important for patients with negative results who still have a concerning family history.

### The Need for Change

The current clinical-criteria/FH-based system of genetic testing has many limitations. It is only moderately effective at identifying mutations and poor at ruling out the presence of one [19]. We [20] and others [21,22] have shown current testing-criteria miss >50% *BRCA*-carriers with a relevant cancer and an even higher proportion of unaffected carriers don’t fulfil current genetic-testing criteria. There are a number of reasons for this, including paternal inheritance, poor communication within and between families, inability to access health records, population migration, smaller nuclear families, lack of awareness and pure chance. In addition, a number of carriers are missed because they will have a probability below the clinical testing threshold (their *BRCA* probability is not nil). Furthermore, the current approach requires individuals to be aware of their FH of cancer, understand its importance, and contact their GP or relevant health professional. The health professional in turn needs to understand the importance of this history and needs to refer to an appropriate genetics centre/clinician. This gate-keeper approach requires people to jump through a number of hoops. Lack of public and health professional awareness and complexity/inefficiency of the current structure and testing pathway has led to restricted access and massive under-utilisation of genetic testing services [23,24]. Childers et al. estimate that >70% BC and >80% OC patients eligible for genetic testing in the USA have never discussed this with a health professional [23]. We recently analysed NHS genetic-laboratory *BRCA*-testing data from 1993–2014 across a Greater-London population of 16 million and found that <3% of estimated *BRCA*-carriers had been identified to date [24]. Our forecasting models suggest detection-rates using the current system are inadequate to identify all *BRCA*-carriers in the population and even doubling them will need 165 years to identify the ‘clinically detectable’ proportion of *BRCA*-carriers (~50% don’t fulfil clinical-testing criteria, remaining undetectable) [24]. Given the small proportion of unaffected individuals getting cancer annually, even addition of unselected case series testing, while useful in identifying the pool of individuals without strong FH of cancer, will require ~250 years to identify residual undetected *BRCA* carriers [24]. Why do we need to wait for decades for people to develop cancer before identifying mutation carriers and their at-risk family members? With the effective options for cancer-risk management and prevention available for high-risk women, this raises serious questions about the adequacy of the current clinical-criteria/FH-based approach. A number of these limitations can be overcome by offering unrestricted/unselected population-based testing. However, adoption of such a strategy should give careful consideration to appropriate results disclosure follow up, particularly if undertaken in the absence of thorough pre-test counseling.

Next-generation sequencing-driven high-throughput testing coupled with advances in bioinformatics has technologically enabled large scale population wide testing. Falling costs of testing and increasing population awareness of cancer genetics and its implications offers a timely opportunity to apply this knowledge and technology on a broad population scale to provide an important impetus in healthcare towards disease prevention. We present a systematic review of the literature on population-based germline testing for *BRCA* gene mutations. We also explore future applicability and potential for this strategy across other CSGs/chronic disease.

## 2. Methods

### 2.1. Search Strategy and Selection Criteria

We systematically reviewed the current literature on population-based germline testing for BRCA-mutations using a comprehensive three-step search strategy to identify relevant studies. First, we searched the following five databases from inception to 30 August 2018: MEDLINE, EMBASE, Pubmed, CINAHL, and PsychINFO. A common search strategy (Table 1) was developed for database searching using a combination of free text and controlled vocabulary (MeSH terms). Second, reference lists of publications retrieved in the first step were screened for relevant studies. Third, we searched additional web-based platforms including specialised journals, Google searches for grey literature, conference proceedings and clinical trial registries (ISRCTN registry/ClinicalTrials.gov registry).

Predefined inclusion criteria were unselected, unaffected individuals at population level risk undergoing genetic-testing for cancer predisposing genes. Outcomes investigated in relation to population genetic testing were: (1) acceptability, (2) testing uptake, (3) mutation detection rate, (4) satisfaction, (5) quality-of-life, (6) psychological health, (7) genetic counselling, (8) knowledge, (9) risk perception and (10) cost-effectiveness.

### 2.2. Data Extraction and Quality Assessment

Data were extracted using a standardised, predesigned data extraction sheet in Microsoft Excel 2013. Four main categories of data were extracted: methodological characteristics of each study, study population, details of interventions and reported outcome measures pertaining to population genetic testing. The quality of the studies was assessed depending on study design, using the following checklists: Quality of Health Economic Studies (QHES) checklist [25], Critical Appraisal Skills Programme (CASP) qualitative research checklist [26], Jadad scale for reporting randomized controlled trials [27] and Methodological Index for Non-Randomized Studies (MINORS) checklist [28].

### 2.3. Data Analysis

We tabulated characteristics and reported outcome measures of all studies for qualitative synthesis.

## 3. Results

**Figure 1:**
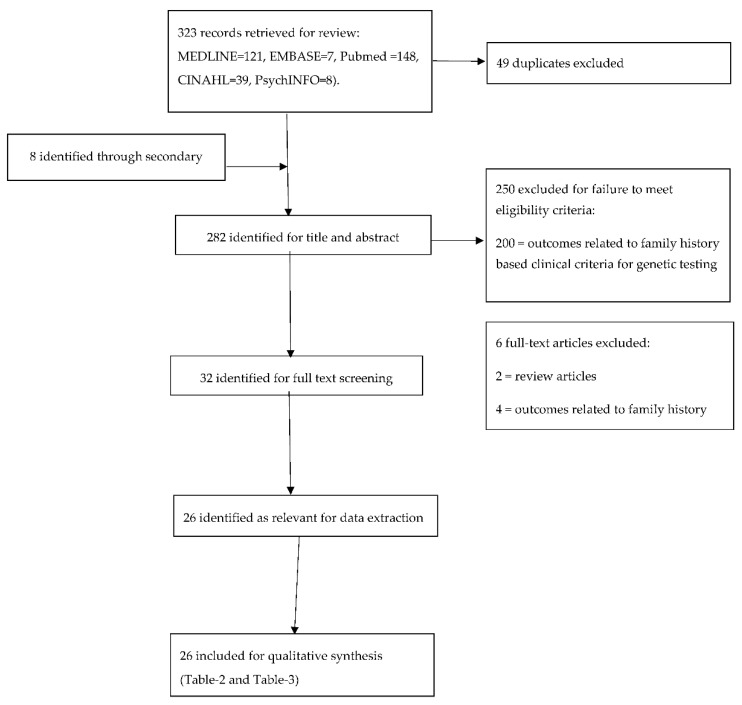
Flowchart of study selection.

Figure 1 provides the flow chart outlining the search outcomes and study selection process. Searches of electronic databases and reference lists generated 323 references. On evaluation of all titles and abstracts, 32/323 articles were potentially eligible for detailed assessment. We found 26/32 met our inclusion criteria for qualitative synthesis [16,20,21,22,29,30,31,32,33,34,35,36,37,38,39,40,41,42,43,44,45,46,47,48,49,50]. Relevant studies on population testing and design/outcomes/quality are summarised in Table 2. Table 3 encapsulates the main findings/conclusion from each study.

### 3.1. The Jewish BRCA Model

The majority of the evidence base for population-based testing currently comes from *BRCA* founder mutation testing (as the genetic disease model) in the Jewish population (population model). Six studies describe attitudes, interest, intention, barriers, and facilitators of *BRCA*-testing in the Ashkenazi Jewish (AJ) population (Table 2 and Table 3) [30,31,39,40,41,45]. Four main studies have evaluated the impact of unselected population-based *BRCA*-testing in the Jewish population: two Israeli cohort studies (8195 men and 1771 women/men) [21,46]; one Canadian cohort study (2080 women) [22]; and one UK randomised controlled trial (RCT) (1034 women and men) [20]. Details of these studies and published outputs are described in Table 2 and Table 3. These studies demonstrate that population-based *BRCA*-testing in the Jewish population is feasible, acceptable, safe, can be undertaken in a community setting, and identifies >50% additional *BRCA*-carriers who would have been missed by traditional clinical-criteria. RCT data show no significant difference in psychological well-being and quality-of-life outcomes between population-based and FH/clinical-criteria-based *BRCA*-testing approaches [20]. Overall anxiety and uncertainty with *BRCA*-testing were found to decrease with time [20]. Israeli and Canadian cohort data show increased anxiety and distress in identified mutation carriers at 6 months/1 year [46,49]. However, overall satisfaction rates are high for all participants (>91%) and similar to non-carriers [46]. Hence, outcomes seen with population-based testing appear to be similar to those reported from high-risk clinics [51].

Both Israeli and UK data suggest testing uptake and satisfaction rates are higher for testing undertaken through self-referral in ambulatory or community centres compared to hospital ascertainment [20,46]. Qualitative data re-confirm overall satisfaction with population-based *BRCA*-testing reported with quantitative analyses, with 81% carriers and 90% non-carriers interviewed expressing unequivocal positive attitudes towards the *BRCA*-testing experience [45]. Barriers and facilitators reported with population-testing are similar to those found in high risk clinics. Other emergent themes reported include the need for incorporating testing into routine practice through primary care and via non-genetic clinicians as well as preservation of autonomy in decision making [45]. Familial communication following testing has been found to be associated with overall satisfaction with the process and FH of cancer. Initial cascade testing rates are higher in first-degree than second-degree relatives [32].

For large-scale, population-based genetic -testing to become feasible/practical it is necessary to move away from the cost and time intensive ‘traditional face-to-face’ genetic-counselling [52] approach. A UK non-inferiority cluster-randomised trial in the Jewish population showed that DVD-assisted pre-test counselling (DVD followed by shorter face-to-face session) for population *BRCA*-testing is an effective, acceptable, non-inferior, time-saving and cost-efficient alternative to traditional 1:1 genetic-counselling [16]. Other studies in high-risk women have established that telephone-counselling is an effective non-inferior alternative to traditional genetic-counselling [14]. The Israeli and Canadian population-based studies successfully undertook *BRCA*-testing without pre-test counselling, and provided post-test counselling. Around 50% of *BRCA*-carriers and 20% of overall participants in the Canadian population-based study expressed a preference for pre-test counselling after receiving their results [49]. Nevertheless, high satisfaction rates (91–95%) are reported in all these (UK/Israeli/Canadian) population-based *BRCA*-testing studies [16,46,49]. Patient satisfaction alone is inadequate for assessing genetic testing alternative delivery models. The UK study also demonstrated non-inferiority for increase in knowledge, risk perception, equivalence for uptake and improvement in understanding of risks/benefits/implications/purpose of PGT. Additionally, other outcomes, including patient empowerment, comfort in decision making and self-efficacy need assessing. A recent UK pilot study has shown acceptability of a web decision-aid plus helpline and post-test counselling approach for population-based testing. Robust RCT data comparing pre-test counselling with decision-aid and helpline or post-test only counselling alone are lacking. Additional research is needed to evaluate genetics service delivery models.

An initial paper confirms the cost-utility of population testing compared to no testing [38]. Three published analyses have evaluated cost-effectiveness of population-based *BRCA*-testing compared to current standard of clinical-criteria/FH testing in: the AJ population [47], the AJ-population with varying AJ-ancestry [48], and the Sephardi Jewish (SJ) population. [50] These show that *BRCA*-testing in the Jewish population is extremely cost-effective compared to FH-based testing. In fact, in most published scenarios the intervention is cost-saving for both UK and USA health systems [48], saving both lives and money. Overall data thus strongly support the introduction of population-based *BRCA*-testing in the Jewish population. It is time guidelines change to reflect this. Multi-gene panel testing has not yet been evaluated in the Jewish population. However, as panel-testing studies evolve this will also become applicable to the Jewish population. Cost-effectiveness for panel testing was recently demonstrated in the general population [33]. As the *BRCA* mutation frequency is higher and costs for next generation sequencing (NGS) similar, this would also be cost-effective in the Jewish population.

The challenge of implementation: There is no single best/ideal model for implementing population-based *BRCA*-testing in the Jewish community. It is likely that different/bespoke models will be needed for various health systems and contexts. Implementation will need development of testing pathways through a community or primary care-based approach outside the traditional hospital-based genetics clinic model, particularly in regions with large or dense Jewish populations. Areas with small or sparse populations could even be absorbed within the current clinical genetics system through changes in testing criteria. Implementation will require significant efforts towards engagement of community leaders, charities, stakeholders, opinion makers and Rabbis across all sections of the community. Additionally, downstream pathways for management of unaffected carriers (including genetics services, gynaecologists, breast clinicians and screening and prevention services) will need expanding or establishing. This will need integration into GP networks to ensure adequate infrastructure and coherent pathways for managing newly identified mutation carriers. This needs to be coupled with information campaigns to increase both public and health professional awareness.

### 3.2. Other Founder Populations

Specific *BRCA* founder mutations have been described in a number of other founder populations (in addition to the Jewish population). These include Polish, French, Swedish, Norwegian, Dutch, Hispanic, Malaysian, Afro-American, Pakistani, Filipino, Inuit and Bahamian populations [53,54,55]. Findings of *BRCA* founder mutation testing studies from the Jewish population could also have implications for *BRCA*-testing in other founder populations. However, it is difficult to currently generalise these beyond this to the rest of the non-founder general population. The Polish ‘Twoj Styl’ study offered Polish *BRCA1* founder mutation testing to 5024 women through a magazine advertisement [56]. Post-test counselling was provided to mutation carriers identified and high satisfaction rates (97%) reported overall. However, this was not ‘true’ unselected population testing as there was ascertainment bias with testing offered only to women with cancer or a FH of breast/ovarian cancer.

### 3.3. General Population and Panel Testing

Next-generation sequencing has enabled testing of multiple CSGs at the same time, i.e., panel testing. This is now being implemented in clinical genetics for women at increased risk fulfilling usual clinical-criteria. Population-based testing can also incorporate multiple genes on an NGS panel. The panel of genes needs to have established analytic validity (sensitivity, specificity, reliability and assay robustness, to reliably and accurately measure the genotype) and clinical validity (test’s ability to reliably and accurately predict the associated disorder/phenotype) [57]. A key unassailable principle underpinning extending panel testing to a population-based setting is only testing for those genes which have well-established ‘clinical utility’, i.e., demonstrable clear net clinical benefit (clinically effective) which can impact disease outcome [57]. A number of genes widely available or offered through panels by gene testing companies/laboratories do not yet have well-established clinical utility. However, the list of genes with proven clinical utility will evolve and expand in the coming years.

A number of other moderate/high-penetrance CSGs (in addition to *BRCA1/BRCA2*) can be incorporated into a population testing panel. Amongst the BC genes, *PALB2* confers non-syndromic quasi-Mendelian susceptibility to BC (BC-risk till age 80 years = 44%) [58], for which equivalent interventions of MRI screening/preventive mastectomy are now offered to mutations carriers and, hence, *PALB2* can be incorporated. Although *ATM* and *CHEK2* are offered on some commercial panels, clinical testing of these genes is not currently routinely undertaken in most centres as the risks conferred by mutations in these genes are moderate (RR~1.5–2). National Comprehensive Cancer Network (NCCN) guidelines support breast screening but mastectomy is not routinely offered for these mutations and family history needs to be incorporated into risk assessment and management. Within the clinical setting, today there remains significant disparity across centers in clinical management available for these mutation carriers. Ascertainment of these (*ATM/CHEK2*) mutations outside the familial context, in individuals from unaffected families, will further amplify uncertainties around penetrance, potentially falling below the threshold for clinical intervention. Hence, these are probably currently best left out of a population-testing panel. As more data become available they may be incorporated into a future population-testing strategy particularly within the context of an overall BC model for risk estimation and population stratification. Amongst the newer moderate risk OC genes, risk estimates for *RAD51C*, *RAD51D* and *BRIP1* (OC-risks ~6–11%) have been recently validated. We showed that surgical prevention (RRSO) is cost-effective at ≥4–5% OC-risk [59,60]. This enables clinical-utility for clinical-testing for these newer moderate OC-risk genes and the option of surgical prevention in unaffected women. Testing for these genes is now incorporated into clinical practice [61] and can be included in a population-based panel. Additionally Lynch-Syndrome (LS) *MLH1/MSH2/MSH6* mismatch-repair (MMR) genes have a 40–60% risk of colorectal cancer, 30–45% risk of Endometrial Cancer (EC) and 6–14% risk of OC [62]. LS/MMR-carriers can benefit from 1–2 yearly colonoscopies for colorectal-cancer screening and opt for daily aspirin [63] or prophylactic hysterectomy-&-oophorectomy for cancer prevention [64]. Amsterdam-II or Bethesda criteria used to identify *MLH1/MHS2/MSH6* carriers in clinical practice miss 55–70% or 12–30% (respectively) of these *MLH1/MHS2/MSH6* carriers [65] even amongst those with cancer. Thus, *MLH1/MHS2/MSH6* are also potential candidate CSGs that can be included in an extended population germline testing panel. Overall these mutations account for around 15–20% OC [66], 6% BC [67], 4–6% EC [68] and 4% bowel-cancers [69].

Initial survey-based data suggest that population-testing for OC gene mutations for risk stratification may be acceptable to 75% women [35], and 72% women anticipate they would engage in positive health behaviour changes in response to BC/OC risk disclosure following genetic testing [36]. An ongoing UK pilot study (ISRCTN54246466) shows feasibility of counselling and recruitment for panel genetic-testing for multiple moderate-high penetrance OC genes in unselected general-population women ascertained through primary care. The team in Toronto have implemented unselected *BRCA* testing for general population Canadian women and men over 18 years who are willing to pay for this themselves, through a Direct to Consumer testing model within ‘The Screen Project’ (http://www.thescreenproject.ca/) study [44]. We recently evaluated the cost-effectiveness of population-based panel testing for OC and BC gene mutations (*BRCA1/BRCA2/RAD51C/RAD51D/BRIP1/PALB2*) by comparing this strategy to the usual clinical-criteria/FH based testing for both UK and US health systems [33]. Modelling showed that population-based panel testing for BC/OC CSGs was more cost-effective than any currently used clinical-criteria/FH-based strategy: either clinical-criteria/FH-based BRCA-testing or clinical-criteria/FH-based panel testing. The ICERs (incremental cost-effectiveness ratios) were well below the thresholds of the UK of £30,000/QALY (ICER = £21,599.96/QALY (quality-adjusted life year)) and the USA of $100,000/QALY (ICER = $54,769.78/QALY). Sensitivity analyses demonstrated that population-testing was the cost-effective and the preferred strategy in 84% of UK and 93% of USA simulations, respectively. This could potentially prevent thousands more BC and OC cases over and above current policy. This was estimated to be 17,505 OC and 64,493 BC cases prevented in UK women, and 65,221 OC and 237,610 BC cases prevented in US women.

However, cost-effectiveness modelling, like all such analyses, incurs assumptions, and further research is necessary for prospective validation of some key assumptions. Jewish data cannot be directly extrapolated or generalised to the non-Jewish general population and general population implementation studies are necessary to evaluate the impact and reconfirm cost-effectiveness of population-based panel testing. More data are needed on uptake rates of screening and prevention options in mutation carriers without a strong FH of cancer. A critical issue which needs addressing is the management of variants of uncertain significance (VUS). Further research is needed around giving VUS results back to individuals, their ability to deal with uncertainty, the impact of this result, developing a robust platform for VUS monitoring and evolving an acceptable long-term management pathway for this.

### 3.4. Return of ‘Incidental’ or ‘Secondary’ Findings of Cancer Gene Mutations in Population Research Studies

Some studies have offered return of incidental or secondary findings of post hoc genetic testing undertaken in patients recruited for other research purposes. Thompson et al. undertook post-hoc genetic testing for *BRCA* mutations in 1997 women and Rowley et al. reported testing in 5908 women over 40 years (mean age 59.2 years) undergoing mammographic screening for BC in the Australian Life-pool study [70,71]. Secondary findings of *BRCA* testing in 50,726 men and women have also been reported through the MyCode Community Health Initiative [72,73]. Preliminary outcomes from such studies show acceptability of returning clinically relevant genetic research results or secondary findings along with engagement with screening/preventive services and are supportive of the concept of broadening access towards a population-based approach. These studies give a good idea of mutation rates. In the 100,000 Genomes Project ‘additional looked-for findings’ are being offered as part of the whole genome analysis (and include 10 cancer-susceptibility genes) [74]. Additionally, in many studies the sub-groups opting for return of incidental/secondary looked-for findings are highly selective and not generalisable to an unselected unaffected general population. For example, the 100,000 Genomes Project is not a true population cohort but comprises individuals with cancer and families with rare paediatric diseases. However, this ‘bolt-on’ paradigm of returning additional secondary findings is very different and not equivalent to prospective uptake of testing CSGs in an unselected unaffected population. Data from these studies cannot be equated to outcomes of impact of true population-based testing. Such an approach does not address in an unbiased and prospective manner key questions of population testing around logistics; information giving, consent and true uptake; VUS management; and subsequent uptake of screening and prevention interventions. These outcomes could potentially be very different when a priori consent is sought for genetic testing for specific clinically actionable gene mutations, compared to vague, less-informed or un-informed consent related to imprecisely defined secondary outcomes in post-hoc research studies.

### 3.5. A Potential Strategy for Chronic Disease Prevention

According to the US Centre for Disease Control and Prevention (CDC), 50% of US adults have ≥1 and 25% of US adults have ≥2 chronic health conditions, and the latter accounts for >90% Medicare expenditure. CDC suggests that chronic diseases and injuries contributed to 2.7 million deaths in 2015 [75]. Corresponding treatment costs and resulting lost productivity amounted to $1.3 trillion. In England, chronic conditions account for 50% of GP appointments, 64% outpatient appointments, 70% inpatient bed days, and 70% of the total health and care spend [76]. The increasing prevalence of long-term/chronic conditions is the biggest challenge facing the UK National Health Service (NHS) [76] and many other health systems. Addressing this is critical to put health systems in a better position to remain viable for the future. The Milken Institute (a non-profit, nonpartisan economic think-tank) has projected that by 2023 if we improved prevention, the US could avoid 40 million cases of chronic disease, cut treatment costs by $220 billion, and increase GDP by $900 billion [77]. According to the CDC-commissioned National Vitals Statistics Reports, the top five causes of death from chronic disease in 2015 were (1) heart disease, (2) cancer, (3) lung disease, (4) accidents and (5) strokes [75]. Many of these can be prevented. WHO estimates that by 2030 the number of deaths due to heart disease, cancer, lung disease, accidents and strokes will rise by 24%, 37%, 32%, 14% and 29% in the Americas and by 23%, 45%, 41%, 23% and 28% worldwide, respectively [78]. As validated disease-specific models for risk prediction improve or develop and evolve, they can be used for population stratification to target the proportion of the population at highest risk of chronic disease. A prime example is cardiovascular disease. Testing for familial hypercholesterolemia could be added to any other genetic testing strategy. In addition, going forward complex models incorporating epidemiological, lifestyle and single nucleotide polymorphism (SNP) data may reach broad mass-based clinical applicability for population stratification and targeted primary prevention. A future population testing programme could target other diseases in addition to cancer. Implementing a new comprehensive population testing strategy can herald a paradigm change in approach which shifts/nudges the needle of healthcare towards prevention.

Addressing the increasing burden of chronic disease poses a major challenge for the future. Different organizations at times give conflicting recommendations which in turn can be exacerbated by the advocacy positions of special interest groups, leading to uncertainty amongst clinicians and inconsistent implementation. Due to increasing time pressures and employers/payers struggling with accelerating health care costs, clinicians may question the value of some preventive interventions. Insurance coverage for individual preventive services, especially new technologies, is inconsistent [79,80]. Public messages conveyed are often inconsistent and increasingly colored by commercial self-interest. Racial and ethnic minorities, socio-economically deprived and other underserved populations have a higher burden of chronic disease and need special attention to reach their full health potential [81]. To this end, it is vital to also address social determinants of health, including economic, social, and geographic factors that influence the health of populations and contribute to chronic diseases and injury.

### 3.6. Population Risk Stratification: Beyond High Penetrance Genes

Newer risk prediction models incorporating validated SNPs (as a polygenic risk score) and epidemiological/clinical factors have improved the precision on individualized risk prediction. This allows division of the population into risk strata, such that the highest risk stratum has a significantly higher risk relative to lower strata, enabling (a) targeted risk-stratified screening and/or (b) targeted prevention for the higher risk strata, as long as the risks of individuals in these strata lie above a well-defined threshold of clinical utility (benefit and effectiveness). It may also identify a low-risk stratum who may benefit for less intense or no screening. This can be useful for making both individualized risk-based decisions and population-based screening or prevention programmes. For example, models have been developed for breast, prostate and ovarian cancer. The Predicting the Risk of Cancer At Screening (PROCAS) study (UKCRN-ID 8080) showed that the addition of SNPs and mammographic breast density to the Tyrer-Cuzick model improves BC risk prediction and could be used for risk-stratified screening in general-population women taking part in a national (NHS) Breast Screening Programme [82]. This was associated with lower anxiety but slightly higher cancer worry than comparison women, with no consistent effect on intention to change behaviour and considerable variation in understanding of test results but high overall satisfaction [83]. The Predicting Risk of Ovarian Malignancy Improved Screening and Early detection Feasibility Study (PROMISE FS) is evaluating the acceptability and feasibility of undertaking a study to stratify an unselected general population on the basis of their predicted lifetime OC-risk, as well as offering risk management options of screening and prevention. The population is stratified into low (<5% OC-risk), intermediate (5–10% OC-risk) and high (>10% OC-risk) risk groups, using a model incorporating SNP based polygenic-risk score, *BRCA1/BRCA2/RAD51C/RAD51D/BIP1* mutations and epidemiological data. Personalised SNP based profiles are also being used for melanoma risk stratification. The SOMBRA (Skin health Online for Melanoma: Better Risk Assessment) RCT, investigates personalised SNP testing for melanoma risk versus un-tested controls [84], in terms of short-term sun protection/self-examination, communication, beliefs, test comprehension/recall, satisfaction and cancer-related distress following testing [84], An Australian pilot RCT (ACTRN12615000356561), evaluated the feasibility and acceptability of communicating personalised SNP derived polygenic-risk scores for melanoma to the public, and its preliminary impact on health behaviour and psychosocial outcomes in 118 individuals [85]. Participants were randomised to intervention (personalised booklet and genetic counselling presenting melanoma polygenic risk) and control (non-personalised educational materials) arms [85]. Results showed no significant difference in behavioural effects, skin cancer-related worry or psychological distress at 3 months [85]. A lot more research is needed to evaluate risk model-based stratified screening and prevention, including implementation studies evaluating clinical effectiveness, impact, cost-effectiveness, health behaviour, psychology, ethical and social consequences.

## 4. Conclusions

Our healthcare structure is currently focused predominantly towards improving diagnosis and treatment of disease rather than illness prevention. The current clinical model for genetic testing is based on FH and serial referral through healthcare services. It requires people to develop cancer before identifying unaffected individuals in the family to target prevention. This process is inefficient, resource intensive and misses a large proportion of individuals or mutation carriers at risk. Population testing can overcome these limitations. The ability to test populations on a large scale is now available, testing costs are falling and the acceptability and awareness of testing is rising. Population-based *BRCA* testing in the Jewish population has been extensively evaluated and found to be acceptable, feasible, clinically effective, safe, associated with high satisfaction rates and cost-effective. There are not many medical interventions that have the potential to save both lives and money, but *BRCA*-testing in the Jewish population is one. Available data support change in guidelines for population-based *BRCA* testing in the Jewish community.

Ongoing studies are evaluating population-based genetic testing for CSGs in the general population. Initial analysis suggests this approach is potentially cost-effective for a panel of BC and OC gene mutations. Serious consideration needs to be given to development, implementation and thorough evaluation of alternative service delivery models for more wide-spread population genetic testing that is linked to genetics experts and downstream management pathways. The increasing appreciation and recognition of the complexities of tumour heterogeneity, tumour evolution and resistant mutations associated with metastatic disease has moderated the initial anticipated impact of precision oncology-driven drug therapy-based approaches. Population testing for established cancer genes can provide an impetus to increase carrier detection rates to maximise prevention and reduce cancer burden. A cancer prevention population-based genetic testing programme can serve as an important model, with programme outputs subsequently informing potential applicability and development of programmes for other chronic diseases. While population testing holds great promise, several challenges need to be addressed for this to materialise. To maximise the impact of population testing a future multi-gene and/or multi-disease panel testing approach/strategy needs to ensure: (A) Clinical utility: Net clinical benefit on disease outcome taking into account benefits and harm of the intervention. (B) Equal access: Ensuring equal access to disease prevention initiatives for all communities regardless of ethnicity, socio-economic background or gender, etc. (C) Broadening research: For effective prevention and eradicating chronic disease it is critical to prioritise high-quality research into disease prevention. There needs to be rebalancing of research funding from diagnosis and treatment towards prevention. For example, only 5% of UK research funding goes into prevention [86]. The impact of panel germline population testing needs to be better understood and evaluated. (D) Robust implementation pathways: These need to be context and health-system/population specific. (E) Cost-effectiveness: Sustainable prevention strategies need to be underpinned by evidence-based approaches that are economically viable and maximise the number of years lived in health. Policy makers and funders need to be educated about the significant cost savings that result from modest increases in prevention funding, and potential savings and increased productivity that can result from prevention promotion by employers, insurers and health funders. (F) Consistent coherent messaging: Public messages need to be consistent (and not be biased or swayed by commercial and vested interests), to increase health professional and public awareness, and to pay special attention to minority, socio-economically deprived and underserved populations or others with higher burden of disease.

## Figures and Tables

**Table 1:** Search strategy for literature search.

Objective	To Identify Published Literature on Unselected Population Based Germline Testing
Data sources	A systematic review of articles with the use of MEDLINE (1946 to August 2018), EMBASE (1974 to August 2018), Pubmed (1996 to August 2018), CINAHL (1937 to August 2018), PsychINFO (1806 to August 2018)
Search strategy	49 searches were undertaken using the below PICO framework: Participants: unaffected men/women Intervention: unselected population genetic testing Comparison: family history/clinical criteria genetic testing Outcomes: acceptability; detection rate; satisfaction; quality of life; cost-effectiveness of unselected genetic testing
1	(LOW RISK).ti,ab
2	exp “LOW RISK”/
3	(POPULATION RISK).ti,ab
4	exp “POPULATION RISK”/
5	1 OR 2 OR 3 OR 4
6	(CANCER).ti,ab
7	exp “CANCER”/
8	6 OR 7
9	(POPULATION GENETIC TESTING).ti,ab
10	exp “POPULATION GENETIC TESTING”/
11	(UNSELECTED GENETIC TESTING).ti,ab
12	exp “UNSELECTED GENETIC TESTING”/
13	9 OR 10 OR 11 OR 12
14	8 AND 13
15	(FAMILY HISTORY).ti,ab
16	exp “FAMILY HISTORY”/
17	15 OR 16
18	(GENETIC TESTING).ti,ab
19	exp “GENETIC TESTING”/
20	18 OR 19
21	8 AND 17 AND 20
22	(BRCA).ti,ab
23	exp “BRCA”/
24	(BRCA AND “1 OR 2”).ti,ab
25	exp “BRCA AND 1 OR 2”/
26	(BRCA AND 1).ti,ab
27	exp “BRCA AND 1”/
28	(BRCA AND 2).ti,ab
29	exp “BRCA AND 2”/
30	22 OR 23 OR 24 OR 25 OR 26 OR 27 OR 28 OR 29
31	8 AND 30
32	14 OR 21 OR 31
33	(ACCEPTABILITY).ti,ab
34	exp “ACCEPTABILITY”/
35	33 OR 34
36	(DETECTION RATE).ti,ab
37	exp “DETECTION RATE”/
38	36 OR 37
39	(SATISFACTION).ti,ab
40	exp “SATISFACTION”/
41	39 OR 40
42	(QUALITY OF LIFE).ti,ab
43	exp “QUALITY OF LIFE”/
44	42 OR 43
45	(COST EFFECTIVE).ti,ab
46	exp “COST EFFECTIVE”/
47	45 OR 46
48	35 OR 38 OR 41 OR 44 OR 47
49	5 AND 32 AND 48
Eligibility criteria	Unselected, unaffected individuals at population level risk undergoing genetic testing for cancer predisposing genes; full text articles in English language.
Data extraction	Citations, abstracts extracted and reviewed by FG. Relevant papers reviewed by authors FG and RM.
Conclusions	Population genetic testing can overcome the limitations of family history/clinical criteria genetic testing. The technology to test populations on a large scale is available and the cost of testing is falling. Population based *BRCA*-testing has been evaluated in the Jewish population and found to be acceptable, clinically effective, safe and cost-saving. However, these data cannot be ‘directly’ extrapolated to the non-Jewish general population. While recent data suggest genetic testing for breast/ovarian cancer gene mutations could also be cost-effective in general population women, additional research, including implementation studies in the general population, are needed to address various knowledge gaps before that step can be considered.

**Table 2:** Publications and registered studies reporting population genetic testing outcomes.

Publication/Registered Study	Country	Sample Size (*n*)	Study Design	Population	Intervention	Outcomes	Follow Up	Quality of Study Methodology
Brown, 1995 [29]	US	N/A	Cost-effectiveness analysis	General population	PGT for *MSH2/MLH1*	Cost per life year gained	N/A	31/100 ^£^
Cousens, 2017 [30]	Australia	370	Prospective, survey	AJ women	Survey on *BRCA1/BRCA2* PGT	Attitudes; acceptability; interest	None	13/16 ^#^
Gabai-Kapara, 2014 [21]	Israel	8195 (& 694 relatives of carriers)	Prospective cohort	AJ men/women	PGT for AJ *BRCA1/BRCA2* founder mutations	Risk of BC/OC in female carriers ascertained through an unaffected male index subject	Not reported	12/16 ^#^
Lehmann, 2002 [31]	US	200	Prospective, survey	AJ women	Telephone survey on *BRCA1/BRCA2* PGT	Attitudes; acceptability	None	12/16 ^#^
Lieberman, 2017 [45]	Israel	36	Qualitative	AJ men/women	Semi structured interviews in individuals undergoing PGT for AJ *BRCA1/BRCA2* founder mutations	Motivators/barriers to testing; satisfaction	18 months	Good ~
Lieberman, 2017 [46]	Israel	1771	Prospective cohort	AJ men/women	PGT for AJ *BRCA1/BRCA2* founder mutations	Uptake; post-test counselling compliance; satisfaction; anxiety; distress; increase in knowledge	6 months	12/16 ^#^
Lieberman, 2018 [32]	Israel	1771	Prospective, cohort	AJ men/women	PGT for AJ *BRCA1/BRCA2* founder mutations	Familial communication; cascade testing	2 years	12/16 ^#^
Manchanda, 2015 [20] (ISRCTN73338115)	UK	1034	Randomised controlled trial	AJ men/women	PGT versus FH based testing of AJ *BRCA1/BRCA2* founder mutations	Acceptability; psychological impact; QoL	3 months	5/5 *
Manchanda, 2015 [47] (ISRCTN73338115)	UK	N/A	Cost-utility analysis	AJ women	PGT versus FH based testing for AJ *BRCA1/BRCA2* founder mutations	Incremental cost effectiveness ratio per quality adjusted life year	N/A	96/100 ^£^
Manchanda, 2016 [16] (ISRCTN73338115)	UK	936	Cluster randomised non-inferiority trial	AJ men/women	DVD assisted versus standard face-to-face pre-test counselling in individuals undergoing PGT of AJ *BRCA1/BRCA2* founder mutations	Uptake; cancer risk perception; increase in knowledge; counselling time; satisfaction	N/A	4/5 *
Manchanda, 2017 [48]	UK, US	N/A	Cost-utility analysis	AJ women	PGT versus FH based testing for AJ *BRCA1/BRCA2* founder mutations with differing AJ ancestry	Incremental cost effectiveness ratio per quality adjusted life year	N/A	90/100 ^£^
Manchanda, 2018 [33]	UK, US	N/A	Cost-utility analysis	General population women	PGT versus FH based testing of *BRCA1/BRCA2/RAD51C/RAD51D/BRIP1/PALB2* mutations	Incremental cost effectiveness ratio per quality adjusted life year	N/A	96/100 ^£^
Meisel, 2016 [34]	UK	829	Prospective, cohort	General population women	Survey	Interest; attitudes	None	12/16 ^#^
Meisel, 2017 [39]	UK	1031	Randomised experimental survey	General population women	Brief information versus lengthier information to inform decision making about participating in a study (PROMISE study) on PGT for OC	Knowledge; intention; attitudes towards taking part in the PROMISE study	None	3/5 *
Meisel, 2017 [36]	UK	837	Cross-sectional survey	General population women	Survey on *BRCA1/BRCA2* PGT	Anticipated health behaviour change; perceived control to disclosure of OC/BC risk	None	11/16 ^#^
Metcalfe, 2010 [22]	Canada	2080	Prospective, cohort	AJ/SJ women	PGT for Jewish *BRCA1/BRCA2* founder mutations	Mutation prevalence	None	14/16 ^#^
Metcalfe, 2010 [49]	Canada	2080	Prospective, cohort	AJ/SJ women	PGT for Jewish *BRCA1/BRCA2* founder mutations	Satisfaction; cancer related distress; cancer risk perception	1 year	14/16 ^#^
Metcalfe, 2012 [37]	Canada	2080	Prospective, cohort	AJ/SJ women	PGT for Jewish *BRCA1/BRCA2* founder mutations	Cancer related distress; uptake of cancer risk reduction options	2 years	14/16 ^#^
Patel, 2018 [50]	UK, US	N/A	Cost-utility analysis	SJ women	PGT versus FH based testing for SJ BRCA1 founder mutations	Incremental cost effectiveness ratio per quality adjusted life year	N/A	90/100 ^£^
Rubinstein, 2009 [38]	US	N/A	Cost-utility analysis	AJ women	PGT for AJ *BRCA1/BRCA2* founder mutations versus ‘no’ genetic testing	Incremental cost effectiveness ratio per quality adjusted life year	N/A	71/100 ^£^
Schwartz, 2001 [39]	US	391	Randomised controlled trial	AJ women	PGT for *BRCA1/BRCA2* educational material versus general BC education control material	Knowledge; perception of risks and limitations; interest	1 month	3/5 *
Shkedi-Rafid, 2012 [40]	Israel	14	Qualitative	Unaffected BRCA1/BRCA2 AJ female carriers ascertained following a positive test result in a male family member who underwent PGT	Semi structured in-depth interviews on PGT for AJ *BRCA1/BRCA2* founder mutations	Emotional implications; motivations; consequences; attitudes	None	Good ^~^
Tang, 2017 [41]	US	243	Cross-sectional survey	Orthodox AJ women	Survey on PGT for *BRCA1/BRCA2*	Knowledge; perceived BC risk/worry; religious/cultural factors affecting decision making	None	13/16 ^#^
Warner, 2005 [42]	Australia	300	Prospective, cohort	AJ men/women	PGT for APC I1307K mutation, but non-disclosure of results	Acceptability; facilitators and barriers to testing	None	10/16 ^#^
PROMISE Feasibility Study [43] (ISRCTN54246466)	UK	100	Prospective, cohort	General population women	PGT for *BRCA1/BRCA2/RAD51C/RAD51D/BRIP1* and subsequent risk stratified screening and prevention	Acceptability; risk perception; cancer worry; QoL; stratification of OC risk; uptake of risk management options; satisfaction/regret; follow up completion rate; telephone helpline use; decision aid use	6 months	N/A
The Screen Project [44]	Canada	10,000	Prospective, cohort	General population men/women	PGT for *BRCA1/BRCA2*	Satisfaction; cancer worry	Not reported	N/A

PGT—population genetic testing; FH—family history; AJ—Ashkenazi Jewish; SJ—Sephardi Jewish; QoL—quality of life; BC—breast cancer; OC—ovarian cancer; PROMISE—Predicting Risk of Ovarian Malignancy Improved Screening and Early detection Feasibility Study; ICER—incremental cost-effective ratio; QALY—quality adjusted life year; ^£^ Quality of study assessed using Quality of Health Economic Studies (QHES) checklist; ˜ Quality of study assessed using the Critical Appraisal Skills Programme (CASP) qualitative research checklist; * Quality of study assessed using the Jadad scale for reporting randomized controlled trials; ^#^ Quality of study assessed using the Methodological Index for Non-Randomized Studies (MINORS) checklist.

**Table 3:** Findings of publications and registered studies reporting population genetic testing outcomes.

Publication/Registered Study	Findings
Brown, 1995 [29]	Exploratory analysis for cost effectiveness of PGT for MMR gene mutations *MLH1/MSH2* compared to FH testing. PGT may be cost-effective if the base case analysis assumes a restrictive set of assumptions most favourable to the outcome with respect to prevalence, costs, clinical efficacy of screening and preventive interventions.
Cousens, 2017 [30]	96.8% support a Jewish *BRCA1/BRCA2* testing program; 65.6% interested in undergoing PGT. Interest in population-based *BRCA*-testing was higher in women <50 years than women >50 years.
Gabai-Kapara, 2014 [21]	For female relatives with *BRCA1/BRCA2* mutations identified through unaffected AJ male relatives, cumulative risk of developing BC/OC by age 60 and 80 respectively were 0.60/0.83 for *BRCA1*; 0.33/0.76 for *BRCA2* carriers. 2.17% AJ carry a *BRCA1/BRCA2* mutation.
Lehmann, 2002 [31]	40% AJ women interested in PGT for *BRCA1/BRCA2*, 40% not interested, and 20% uncertain. Increased interest associated with desire to obtain information on children’s risk and valuing information for its own sake. 17% expressed concern or discomfort about Jews being offered *BRCA1/2* testing. Increased concern about genetic discrimination associated with highly educated women.
Lieberman, 2017 [45]	Motivators for *BRCA* testing: knowledge of *BRCA* status to enable cancer risk reduction; health-empowerment. Barriers: lack of physician awareness/support. Routinization of testing can overcome medical and social barriers. Importance of maintaining/safeguarding autonomy of choice and providing adequate post-test services was highlighted.
Lieberman, 2017 [46]	*BRCA* testing uptake 67%. Post-test counselling compliance 100% for carriers; 89% for non-carriers with FH. All groups had high satisfaction (>90%). At 6 months, carriers had significantly increased distress/anxiety; greater knowledge; similar satisfaction to non-carriers. 90% recommended PGT for *BRCA* in the AJ community. Proactive recruitment through a clinical service captured older women more unselected for FH compared to self-referral based recruitment.
Lieberman, 2018 [32]	97% carriers informed at least one relative. FH and higher Satisfaction With Health Decision scores predicted results communication. FDRs had a higher rate of cascade/predictive testing than SDRs. Female relatives had a higher level of cascade testing than male relatives.
Manchanda, 2015 [20] (ISRCTN73338115)	Compared with FH based testing, PGT for *BRCA1/BRCA2* AJ founder mutations, does not adversely affect short-term psychological/QoL outcomes and may detect 56% additional BRCA carriers. 56% of carriers do not fulfil clinical criteria for genetic testing, and the *BRCA1/2* prevalence is 2.45%.
Manchanda, 2015 [47] (ISRCTN73338115)	PGT for AJ *BRCA1/BRCA2* founder mutations is cost saving with a baseline discounted ICER of -£2079/QALY. PGT lowered OC/BC incidence by 0.34% and 0.62% respectively. Assuming 71% testing uptake, this leads to 276 fewer OC and 508 fewer BC cases. Overall, reduction in treatment costs leads to a discounted cost savings of £3.7 million in the UK population.
Manchanda, 2016 [16] (ISRCTN73338115)	DVD-assisted counselling for PGT is non-inferior to face-to-face counselling for increase in knowledge; counselling satisfaction; risk perception and is equivalent for uptake. 98% found DVD length/information satisfactory. 85–89% felt it improved understanding of risks/benefits/implications/purpose of PGT. 95% would recommend it to others.
Manchanda, 2017 [48]	PGT for *BRCA* mutations is cost-saving in AJ with 2–4 grandparents (22–33 days life gained) in the UK and 1–4 grandparents (12–26 days life-gained) in the US. It is extremely cost-effective in women in the UK with 1 AJ grandparent with ICER = £863/QALY; 15 days life gained. PGT remains cost-effective in the absence of reduction in BC risk from RRSO; at lower RRM (13%) or RRSO (20%) rates.
Manchanda, 2018 [33]	Population panel genetic testing for *BRCA1/BRCA2/RAD51C/RAD51D/BRIP1/PALB2* mutations is the most cost-effective genetic testing strategy compared with current policy: ICER = £21,599.96/QALY or $54,769.78/QALY (9.34 or 7.57 days’ life-expectancy gained). PGT for *BRCA1/BRCA2/RAD51C/RAD51D/BRIP1/PALB2* testing can prevent 1.86%/1.91% of BC and 3.2%/4.88% of OC in UK/US women: 657/655 OC cases and 2420/2386 BC cases prevented per million.
Meisel, 2016 [34]	85% reported they would ‘probably’ or ‘definitely’ take up PGT for OC which increased to 88% if test also informed BC risk. 92% anticipated they would ‘probably’ or ‘definitely’ participate in risk-stratified OC screening. University level education is associated with lower anticipated uptake of PGT.
Meisel, 2017 [39]	No significant differences between participants receiving brief versus lengthier information to inform decision making in terms of OC knowledge/intention to participate in OC screening following PGT. 74% reported they would participate in OC screening based on PGT assessment.
Meisel, 2017 [36]	UK women anticipate that they would engage in positive health behaviour changes in response to BC/OC risk disclosure. 72% reported ‘I would try harder to have a healthy lifestyle’; 55% felt ‘it would give me more control over my life’. Associations were independent of demographic factors or perceived risk of OC/BC.
Metcalfe, 2010 [22]	Overall *BRCA1/BRCA2* prevalence in unselected Jewish women undergoing PGT was 1.1% (0.5% for *BRCA1* and 0.6% for *BRCA2*). Only 45% met clinical testing criteria.
Metcalfe, 2010 [49]	In Jewish *BRCA* carriers, mean BC risk perception increased significantly from 41.1% to 59.6% after receiving a positive result. Among non-carriers, BC risk perception decreased non-significantly, from 35.8% to 33.5%. Cancer-related distress increased significantly for carriers, but not in non-carriers. 92.8% satisfied with PGT.
Metcalfe, 2012 [37]	Within 2 years of receiving a positive Jewish *BRCA* founder mutation result, 11.1% had RRM; 89.5% RRSO. Mean BC risk estimated to be 37.2% at time of testing versus 20.9% at 2 years post-testing. Distress decreased between 1 and 2 years for women with RRM/RRSO and for women with only RRSO but not for those with no surgery.
Patel, 2018 [50]	PGT is cost-effective for SJ *BRCA1* founder mutation. It results in 12 months (QALY = 1.00) gain in life expectancy. Baseline discounted ICER for UK PGT = £67.04/QALY; US population = $308.42/QALY. PGT remains cost effective in UK/US, even if premenopausal RRSO doesn’t reduce BC risk or if HRT compliance is nil.
Rubinstein, 2009 [38]	Compared to a no testing policy, PGT for AJ *BRCA1/BRCA2* founder mutations is cost-effective and would result in 2811 fewer cases of OC, with a life expectancy gain of 1.83 QALYs among carriers. At a cost of $460 for founder mutation testing, the cost of the program is $8300/QALY.
Schwartz, 2001 [39]	Compared to the BC education control material, the PGT education material led to increased knowledge; increased perception of the risks/limitations of testing; and a decreased interest in obtaining a *BRCA1/BRCA2* test.
Shkedi-Rafid, 2012 [40]	Having no FH of cancer was a source of optimism but also confusion; engaging in intensified medical surveillance and undergoing preventive procedures was perceived as health promoting but also induced a sense of physical/psychological vulnerability; overall support for population *BRCA*-testing in the AJ community, with some reservations.
Tang, 2017 [41]	49% had adequate genetic testing knowledge; 46% had accurate BC risk perceptions. 20% reported they probably/definitely will get tested; 28% probably/definitely will not get tested; 46% had not thought about *BRCA* testing. Adequate genetic testing knowledge, higher BC risk, and overestimation of risk is associated with PGT intention. Cancer prevention and effect on children were the most important factors affecting testing intention.
Warner, 2005 [42]	Following pre-test counselling 94% acceptability for PGT for colorectal cancer, but participants were not disclosed results. Facilitators: desire for information for their families; to decrease personal cancer risk. Barriers: insurance discrimination; test accuracy; confidentiality.
PROMISE Feasibility Study [43] (ISRCTN54246466)	Not reported. Study closed to recruitment and in follow up phase.
The Screen Project	Not reported. Study actively recruiting.

PGT—population genetic testing; FH—family history; AJ—Ashkenazi Jewish; QoL—quality of life; BC—breast cancer; OC—ovarian cancer; FDR—first degree relative; SDR—second degree relative; ICER—incremental cost-effective ratio; QALY—quality adjusted life year.

## References

[1] Kuchenbaecker K.B., Hopper J.L., Barnes D.R., Phillips K.A., Mooij T.M., Roos-Blom M.J., Jervis S., van Leeuwen F.E., Milne R.L., Andrieu N. (2017). Risks of Breast, Ovarian, and Contralateral Breast Cancer for BRCA1 and BRCA2 Mutation Carriers. JAMA.

[2] Rebbeck T.R., Kauff N.D., Domchek S.M. (2009). Meta-analysis of risk reduction estimates associated with risk-reducing salpingo-oophorectomy in BRCA1 or BRCA2 mutation carriers. J. Natl. Cancer Inst..

[3] Cuzick J., Sestak I., Bonanni B., Costantino J.P., Cummings S., DeCensi A., Dowsett M., Forbes J.F., Ford L., LaCroix A.Z. (2013). Selective oestrogen receptor modulators in prevention of breast cancer: An updated meta-analysis of individual participant data. Lancet.

[4] Rebbeck T.R., Friebel T., Lynch H.T., Neuhausen S.L., van’t Veer L., Garber J.E., Evans G.R., Narod S.A., Isaacs C., Matloff E. (2004). Bilateral prophylactic mastectomy reduces breast cancer risk in BRCA1 and BRCA2 mutation carriers: the PROSE Study Group. J. Clin. Oncol..

[5] Menon U., Harper J., Sharma A., Fraser L., Burnell M., Elmasry K., Rodeck C., Jacobs I. (2007). Views of BRCA gene mutation carriers on preimplantation genetic diagnosis as a reproductive option for hereditary breast and ovarian cancer. Hum. Reprod..

[6] Morgan R.D., Clamp A.R., Evans D.G.R., Edmondson R.J., Jayson G.C. (2018). PARP inhibitors in platinum-sensitive high-grade serous ovarian cancer. Cancer Chemother. Pharmacol..

[7] Ison G., Howie L.J., Amiri-Kordestani L., Zhang L., Tang S., Sridhara R., Pierre V., Charlab R., Ramamoorthy A., Song P. (2018). FDA Approval Summary: Niraparib for the Maintenance Treatment of Patients with Recurrent Ovarian Cancer in Response to Platinum-Based Chemotherapy. Clin. Cancer Res..

[8] Coleman R.L., Oza A.M., Lorusso D., Aghajanian C., Oaknin A., Dean A., Colombo N., Weberpals J.I., Clamp A., Scambia G. (2017). Rucaparib maintenance treatment for recurrent ovarian carcinoma after response to platinum therapy (ARIEL3): A randomised, double-blind, placebo-controlled, phase 3 trial. Lancet.

[9] Ledermann J., Harter P., Gourley C., Friedlander M., Vergote I., Rustin G., Scott C.L., Meier W., Shapira-Frommer R., Safra T. (2014). Olaparib maintenance therapy in patients with platinum-sensitive relapsed serous ovarian cancer: A preplanned retrospective analysis of outcomes by BRCA status in a randomised phase 2 trial. Lancet Oncol..

[10] American Society of Clinical Oncology (2003). American Society of Clinical Oncology policy statement update: Genetic testing for cancer susceptibility. J. Clin. Oncol..

[11] International Huntington Association and the World Federation of Neurology Research Group on Huntington’s Chorea (1994). Guidelines for the molecular genetics predictive test in Huntington’s disease. J. Med. Genet..

[12] Calzone K.A., Prindiville S.A., Jourkiv O., Jenkins J., DeCarvalho M., Wallerstedt D.B., Liewehr D.J., Steinberg S.M., Soballe P.W., Lipkowitz S. (2005). Randomized comparison of group versus individual genetic education and counseling for familial breast and/or ovarian cancer. J. Clin. Oncol..

[13] Jenkins J., Calzone K.A., Dimond E., Liewehr D.J., Steinberg S.M., Jourkiv O., Klein P., Soballe P.W., Prindiville S.A., Kirsch I.R. (2007). Randomized comparison of phone versus in-person BRCA1/2 predisposition genetic test result disclosure counseling. Genet. Med..

[14] Kinney A.Y., Butler K.M., Schwartz M.D., Mandelblatt J.S., Boucher K.M., Pappas L.M., Gammon A., Kohlmann W., Edwards S.L., Stroup A.M. (2014). Expanding access to BRCA1/2 genetic counseling with telephone delivery: a cluster randomized trial. J. Natl. Cancer Inst..

[15] Kinney A.Y., Steffen L.E., Brumbach B.H., Kohlmann W., Du R., Lee J.H., Gammon A., Butler K., Buys S.S., Stroup A.M. (2016). Randomized Noninferiority Trial of Telephone Delivery of BRCA1/2 Genetic Counseling Compared With In-Person Counseling: 1-Year Follow-Up. J. Clin. Oncol..

[16] Manchanda R., Burnell M., Loggenberg K., Desai R., Wardle J., Sanderson S.C., Gessler S., Side L., Balogun N., Kumar A. (2016). Cluster-randomised non-inferiority trial comparing DVD-assisted and traditional genetic counselling in systematic population testing for BRCA1/2 mutations. J. Med. Genet..

[17] Schwartz M.D., Valdimarsdottir H.B., Peshkin B.N., Mandelblatt J., Nusbaum R., Huang A.T., Chang Y., Graves K., Isaacs C., Wood M. (2014). Randomized noninferiority trial of telephone versus in-person genetic counseling for hereditary breast and ovarian cancer. J. Clin. Oncol..

[18] George A., Riddell D., Seal S., Talukdar S., Mahamdallie S., Ruark E., Cloke V., Slade I., Kemp Z., Gore M. (2016). Implementing rapid, robust, cost-effective, patient-centred, routine genetic testing in ovarian cancer patients. Sci. Rep..

[19] Kang H.H., Williams R., Leary J., Ringland C., Kirk J., Ward R. (2006). Evaluation of models to predict BRCA germline mutations. Br. J. Cancer.

[20] Manchanda R., Loggenberg K., Sanderson S., Burnell M., Wardle J., Gessler S., Side L., Balogun N., Desai R., Kumar A. (2015). Population testing for cancer predisposing BRCA1/BRCA2 mutations in the Ashkenazi-Jewish community: A randomized controlled trial. J. Natl. Cancer Inst..

[21] Gabai-Kapara E., Lahad A., Kaufman B., Friedman E., Segev S., Renbaum P., Beeri R., Gal M., Grinshpun-Cohen J., Djemal K. (2014). Population-based screening for breast and ovarian cancer risk due to BRCA1 and BRCA2. Proc. Natl. Acad. Sci. USA.

[22] Metcalfe K.A., Poll A., Royer R., Llacuachaqui M., Tulman A., Sun P., Narod S.A. (2010). Screening for founder mutations in BRCA1 and BRCA2 in unselected Jewish women. J. Clin. Oncol..

[23] Childers C.P., Childers K.K., Maggard-Gibbons M., Macinko J. (2017). National Estimates of Genetic Testing in Women With a History of Breast or Ovarian Cancer. J. Clin. Oncol..

[24] Manchanda R., Blyuss O., Gaba F., Gordeev V.S., Jacobs C., Burnell M., Gan C., Taylor R., Turnbull C., Legood R. (2018). Current detection rates and time-to-detection of all identifiable BRCA carriers in the Greater London population. J. Med. Genet..

[25] Chiou C.F., Hay J.W., Wallace J.F., Bloom B.S., Neumann P.J., Sullivan S.D., Yu H.T., Keeler E.B., Henning J.M., Ofman J.J. (2003). Development and validation of a grading system for the quality of cost-effectiveness studies. Med. Care.

[26] (2018). Critical Appraisal Skills Programme. CASP Qualitative Checklist.

[27] Clark H.D., Wells G.A., Huet C., McAlister F.A., Salmi L.R., Fergusson D., Laupacis A. (1999). Assessing the quality of randomized trials: Reliability of the Jadad scale. Control Clin. Trials.

[28] Slim K., Nini E., Forestier D., Kwiatkowski F., Panis Y., Chipponi J. (2003). Methodological index for non-randomized studies (minors): Development and validation of a new instrument. ANZ J. Surg.

[29] Brown M.L., Kessler L.G. (1995). The use of gene tests to detect hereditary predisposition to cancer: economic considerations. JNCI.

[30] Cousens N., Kaur R., Meiser B., Andrews L. (2017). Community attitudes towards a Jewish community BRCA1/2 testing program. Fam. Cancer.

[31] Lehmann L.S., Weeks J.C., Klar N., Garber J.E. (2002). A population-based study of Ashkenazi Jewish women’s attitudes toward genetic discrimination and BRCA1/2 testing. Genet. Med..

[32] Lieberman S., Lahad A., Tomer A., Koka S., BenUziyahu M., Raz A., Levy-Lahad E. (2018). Familial communication and cascade testing among relatives of BRCA population screening participants. Genet. Med..

[33] Manchanda R., Patel S., Gordeev V.S., Antoniou A.C., Smith S., Lee A., Hopper J.L., MacInnis R.J., Turnbull C., Ramus S.J. (2018). Cost-effectiveness of Population-Based BRCA1, BRCA2, RAD51C, RAD51D, BRIP1, PALB2 Mutation Testing in Unselected General Population Women. J. Natl. Cancer Inst..

[34] Meisel S.F., Rahman B., Side L., Fraser L., Gessler S., Lanceley A., Wardle J., PROMISE-2016 study team (2016). Genetic testing and personalized ovarian cancer screening: a survey of public attitudes. BMC Womens Health.

[35] Meisel S.F., Freeman M., Waller J., Fraser L., Gessler S., Jacobs I., Kalsi J., Manchanda R., Rahman B., Side L. (2017). Impact of a decision aid about stratified ovarian cancer risk-management on women’s knowledge and intentions: A randomised online experimental survey study. BMC Public Health.

[36] Meisel S.F., Fraser L.S.M., Side L., Gessler S., Hann K.E.J., Wardle J., Lanceley A., PROMISE study team (2017). Anticipated health behaviour changes and perceived control in response to disclosure of genetic risk of breast and ovarian cancer: A quantitative survey study among women in the UK. BMJ Open.

[37] Metcalfe K.A., Mian N., Enmore M., Poll A., Llacuachaqui M., Nanda S., Sun P., Hughes K.S., Narod S.A. (2012). Long-term follow-up of Jewish women with a BRCA1 and BRCA2 mutation who underwent population genetic screening. Breast Cancer Res. Treat..

[38] Rubinstein W.S., Jiang H., Dellefave L., Rademaker A.W. (2009). Cost-effectiveness of population-based BRCA1/2 testing and ovarian cancer prevention for Ashkenazi Jews: A call for dialogue. Genet. Med..

[39] Schwartz M.D., Benkendorf J., Lerman C., Isaacs C., Ryan-Robertson A., Johnson L. (2001). Impact of educational print materials on knowledge, attitudes, and interest in BRCA1/BRCA2: Testing among Ashkenazi Jewish women. Cancer.

[40] Shkedi-Rafid S., Gabai-Kapara E., Grinshpun-Cohen J., Levy-Lahad E. (2012). BRCA genetic testing of individuals from families with low prevalence of cancer: Experiences of carriers and implications for population screening. Genet. Med..

[41] Tang E.Y., Trivedi M.S., Kukafka R., Chung W.K., David R., Respler L., Leifer S., Schechter I., Crew K.D. (2017). Population-Based Study of Attitudes toward BRCA Genetic Testing among Orthodox Jewish Women. Breast J..

[42] Warner B.J., Curnow L.J., Polglase A.L., Debinski H.S. (2005). Factors influencing uptake of genetic testing for colorectal cancer risk in an Australian Jewish population. J. Genet. Couns..

[43] Manchanda R. Predicting Risk of ovarian Malignancy Improved Screening and Early Detection Feasibility Study ISRCTN Registry: ISRCTN54246466. 2017. http://www.isrctn.com/ISRCTN54246466.

[44] The Screen Project. http://thescreenproject.ca/.

[45] Lieberman S., Lahad A., Tomer A., Cohen C., Levy-Lahad E., Raz A. (2016). Population screening for BRCA1/BRCA2 mutations: Lessons from qualitative analysis of the screening experience. Genet. Med..

[46] Lieberman S., Tomer A., Ben-Chetrit A., Olsha O., Strano S., Beeri R., Koka S., Fridman H., Djemal K., Glick I. (2016). Population screening for BRCA1/BRCA2 founder mutations in Ashkenazi Jews: Proactive recruitment compared with self-referral. Genet. Med..

[47] Manchanda R., Legood R., Burnell M., McGuire A., Raikou M., Loggenberg K., Wardle J., Sanderson S., Gessler S., Side L. (2015). Cost-effectiveness of population screening for BRCA mutations in Ashkenazi Jewish women compared with family history-based testing. J. Natl. Cancer Inst..

[48] Manchanda R., Patel S., Antoniou A.C., Levy-Lahad E., Turnbull C., Evans D.G., Hopper J.L., Macinnis R.J., Menon U., Jacobs I. (2017). Cost-effectiveness of population based BRCA testing with varying Ashkenazi Jewish ancestry. Am. J. Obstet. Gynecol..

[49] Metcalfe K.A., Poll A., Llacuachaqui M., Nanda S., Tulman A., Mian N., Sun P., Narod S.A. (2010). Patient satisfaction and cancer-related distress among unselected Jewish women undergoing genetic testing for BRCA1 and BRCA2. Clin. Genet..

[50] Patel S., Legood R., Evans D.G., Turnbull C., Antoniou A.C., Menon U., Jacobs I., Manchanda R. (2018). Cost effectiveness of population based BRCA1 founder mutation testing in Sephardi Jewish women. Am. J. Obstet. Gynecol..

[51] Nelson H.D., Fu R., Goddard K., Mitchell J.P., Okinaka-Hu L., Pappas M., Zakher B. (2013). Risk Assessment, Genetic Counseling, and Genetic Testing for BRCA-Related Cancer: Systematic Review to Update the U.S..

[52] Nelson H.D., Huffman L.H., Fu R., Harris E.L. (2005). Genetic risk assessment and BRCA mutation testing for breast and ovarian cancer susceptibility: Systematic evidence review for the U.S. Preventive Services Task Force. Ann. Intern. Med..

[53] Ferla R., Calo V., Cascio S., Rinaldi G., Badalamenti G., Carreca I., Surmacz E., Colucci G., Bazan V., Russo A. (2007). Founder mutations in BRCA1 and BRCA2 genes. Ann. Oncol..

[54] Trottier M., Lunn J., Butler R., Curling D., Turnquest T., Francis W., Halliday D., Royer R., Zhang S., Li S. (2016). Prevalence of founder mutations in the BRCA1 and BRCA2 genes among unaffected women from the Bahamas. Clin. Genet..

[55] Harboe T.L., Eiberg H., Kern P., Ejlertsen B., Nedergaard L., Timmermans-Wielenga V., Nielsen I.M., Bisgaard M.L. (2009). A high frequent BRCA1 founder mutation identified in the Greenlandic population. Fam. Cancer.

[56] Gronwald J., Huzarski T., Byrski T., Debniak T., Metcalfe K., Narod S.A., Lubinski J. (2006). Direct-to-patient BRCA1 testing: The Twoj Styl experience. Breast Cancer Res. Treat..

[57] Teutsch S.M., Bradley L.A., Palomaki G.E., Haddow J.E., Piper M., Calonge N., Dotson W.D., Douglas M.P., Berg A.O., Group E.W. (2009). The Evaluation of Genomic Applications in Practice and Prevention (EGAPP) Initiative: Methods of the EGAPP Working Group. Genet. Med..

[58] Antoniou A.C., Casadei S., Heikkinen T., Barrowdale D., Pylkas K., Roberts J., Lee A., Subramanian D., De Leeneer K., Fostira F. (2014). Breast-cancer risk in families with mutations in PALB2. N. Engl. J. Med..

[59] Manchanda R., Legood R., Antoniou A.C., Gordeev V.S., Menon U. (2016). Specifying the ovarian cancer risk threshold of ’premenopausal risk-reducing salpingo-oophorectomy’ for ovarian cancer prevention: A cost-effectiveness analysis. J. Med. Genet..

[60] Manchanda R., Legood R., Pearce L., Menon U. (2015). Defining the risk threshold for risk reducing salpingo-oophorectomy for ovarian cancer prevention in low risk postmenopausal women. Gynecol. Oncol..

[61] Manchanda R., Menon U. (2018). Setting the Threshold for Surgical Prevention in Women at Increased Risk of Ovarian Cancer. Int. J. Gynecol. Cancer.

[62] Barrow E., Hill J., Evans D.G. (2013). Cancer risk in Lynch Syndrome. Fam. Cancer.

[63] Burn J., Gerdes A.M., Macrae F., Mecklin J.P., Moeslein G., Olschwang S., Eccles D., Evans D.G., Maher E.R., Bertario L. (2011). Long-term effect of aspirin on cancer risk in carriers of hereditary colorectal cancer: An analysis from the CAPP2 randomised controlled trial. Lancet.

[64] Vasen H.F., Blanco I., Aktan-Collan K., Gopie J.P., Alonso A., Aretz S., Bernstein I., Bertario L., Burn J., Capella G. (2013). Revised guidelines for the clinical management of Lynch syndrome (HNPCC): recommendations by a group of European experts. Gut.

[65] (2014). ACOG Practice Bulletin No. 147: Lynch syndrome. Obstet. Gynecol..

[66] Harter P., Hauke J., Heitz F., Reuss A., Kommoss S., Marme F., Heimbach A., Prieske K., Richters L., Burges A. (2017). Prevalence of deleterious germline variants in risk genes including BRCA1/2 in consecutive ovarian cancer patients (AGO-TR-1). PLoS ONE.

[67] Buys S.S., Sandbach J.F., Gammon A., Patel G., Kidd J., Brown K.L., Sharma L., Saam J., Lancaster J., Daly M.B. (2017). A study of over 35,000 women with breast cancer tested with a 25-gene panel of hereditary cancer genes. Cancer.

[68] Ferguson S.E., Aronson M., Pollett A., Eiriksson L.R., Oza A.M., Gallinger S., Lerner-Ellis J., Alvandi Z., Bernardini M.Q., MacKay H.J. (2014). Performance characteristics of screening strategies for Lynch syndrome in unselected women with newly diagnosed endometrial cancer who have undergone universal germline mutation testing. Cancer.

[69] Hampel H., Frankel W.L., Martin E., Arnold M., Khanduja K., Kuebler P., Clendenning M., Sotamaa K., Prior T., Westman J.A. (2008). Feasibility of screening for Lynch syndrome among patients with colorectal cancer. J. Clin. Oncol..

[70] Thompson E.R., Rowley S.M., Li N., McInerny S., Devereux L., Wong-Brown M.W., Trainer A.H., Mitchell G., Scott R.J., James P.A. (2016). Panel Testing for Familial Breast Cancer: Calibrating the Tension Between Research and Clinical Care. J. Clin. Oncol..

[71] Rowley S.M., Mascarenhas L., Devereux L., Li N., Amarasinghe K.C., Zethoven M., Lee J.E.A., Lewis A., Morgan J.A., Limb S. (2018). Population-based genetic testing of asymptomatic women for breast and ovarian cancer susceptibility. Genet. Med..

[72] Buchanan A.H., Manickam K., Meyer M.N., Wagner J.K., Hallquist M.L.G., Williams J.L., Rahm A.K., Williams M.S., Chen Z.E., Shah C.K. (2018). Early cancer diagnoses through BRCA1/2 screening of unselected adult biobank participants. Genet. Med..

[73] Schwartz M.L.B., McCormick C.Z., Lazzeri A.L., Lindbuchler D.M., Hallquist M.L.G., Manickam K., Buchanan A.H., Rahm A.K., Giovanni M.A., Frisbie L. (2018). A Model for Genome-First Care: Returning Secondary Genomic Findings to Participants and Their Healthcare Providers in a Large Research Cohort. Am. J. Hum. Genet..

[74] Turnbull C., Scott R.H., Thomas E., Jones L., Murugaesu N., Pretty F.B., Halai D., Baple E., Craig C., Hamblin A. (2018). The 100 000 Genomes Project: Bringing whole genome sequencing to the NHS. BMJ.

[75] Murphy S.L., Xu J.Q., Kochanek K.D., Curtin S.C., Arias E. Deaths: Final Data for 2015. https://www.cdc.gov/nchs/data/nvsr/nvsr66/nvsr66_06.pdf.

[76] Department of Health Long Term Conditions Team (2012). Long Term Conditions Compendium of Information.

[77] (2014). Checkup Time: Chronic Disease and Wellness in America.

[78] WHO Projections of mortality and causes of death, 2015 and 2030. http://www.who.int/healthinfo/global_burden_disease/projections/en/.

[79] Borry P., Stultiens L., Goffin T., Nys H., Dierickx K. (2008). Minors and informed consent in carrier testing: A survey of European clinical geneticists. J. Med. Ethics.

[80] Shiri-Sverdlov R., Oefner P., Green L., Baruch R.G., Wagner T., Kruglikova A., Haitchick S., Hofstra R.M., Papa M.Z., Mulder I. (2000). Mutational analyses of BRCA1 and BRCA2 in Ashkenazi and non-Ashkenazi Jewish women with familial breast and ovarian cancer. Hum. Mutat..

[81] Ganguly T., Dhulipala R., Godmilow L., Ganguly A. (1998). High throughput fluorescence-based conformation-sensitive gel electrophoresis (F-CSGE) identifies six unique BRCA2 mutations and an overall low incidence of BRCA2 mutations in high-risk BRCA1-negative breast cancer families. Hum. Genet..

[82] Evans D.G., Astley S., Stavrinos P., Harkness E., Donnelly L.S., Dawe S., Jacob I., Harvie M., Cuzick J., Brentnall A. (2016). Improvement in risk prediction, early detection and prevention of breast cancer in the NHS Breast Screening Programme and family history clinics: A dual cohort study. Programme Grants for Applied Research.

[83] French D.P., Southworth J., Howell A., Harvie M., Stavrinos P., Watterson D., Sampson S., Evans D.G., Donnelly L.S. (2018). Psychological impact of providing women with personalised 10-year breast cancer risk estimates. Br. J. Cancer.

[84] Hay J.L., Berwick M., Zielaskowski K., White K.A., Rodríguez V.M., Robers E., Guest D.D., Sussman A., Talamantes Y., Schwartz M.R. (2017). Implementing an Internet-Delivered Skin Cancer Genetic Testing Intervention to Improve Sun Protection Behavior in a Diverse Population: Protocol for a Randomized Controlled Trial. JMIR Res. Protoc..

[85] Smit A.K., Espinoza D., Newson A.J., Morton R.L., Fenton G., Freeman L., Dunlop K., Butow P.N., Law M.H., Kimlin M.G. (2017). A Pilot Randomized Controlled Trial of the Feasibility, Acceptability, and Impact of Giving Information on Personalized Genomic Risk of Melanoma to the Public. Cancer Epidemiol. Biomarkers Prev..

[86] NCRI (2017). NCRI Partners’ Research Spend in 2016.

